# Choroidal Morphology and Systemic Circulation Changes During the Menstrual Cycle in Healthy Japanese Women

**DOI:** 10.7759/cureus.48124

**Published:** 2023-11-01

**Authors:** Kana Kurahashi, Yuki Hashimoto, Kanon Shiraishi, Nonoka Kubota, Takeshi Yoshitomi

**Affiliations:** 1 Department of Orthoptics, Fukuoka International University of Health and Welfare, Fukuoka, JPN

**Keywords:** blood pressure, subfoveal choroidal thickness, enhanced depth imaging optical coherence tomography, menstrual cycle, systemic circulation, choroidal morphology

## Abstract

Purpose: Changes in systemic circulatory dynamics and choroidal thickness are poorly understood. This study aimed to investigate the time course of changes in choroidal morphology during normal menstrual cycles in healthy women using enhanced depth imaging optical coherence tomography (EDI-OCT).

Materials and Methods: This prospective study included 15 left eyes of 15 healthy Japanese women (mean age, 20.2 ± 0.8 years) with a normal menstrual cycle. Using EDI-OCT, the subfoveal choroidal thickness (SCT) was manually measured during the late follicular and mid-luteal phases. Intraocular pressure (IOP), systolic, diastolic, and mean blood pressure (SBP, DBP, and MBP), and heart rate (HR) were also evaluated during these phases.

Results: SBP, DBP, and MBP were significantly elevated in the mid-luteal phase. The average SCT was significantly decreased in the mid-luteal phase. In contrast, there were no significant changes in IOP or HR.

Conclusion: These findings indicate that choroidal thickness decreases during the mid-luteal phase in healthy Japanese women with normal menstrual cycles depending on systemic circulatory dynamics. However, since the difference in the SCT values between the late follicular and the mid-luteal phase is not large (7 μm), the menstrual cycle may have little influence on the interpretation of choroidal thickness data in clinical practice.

## Introduction

The four phases of the menstrual cycle are menstruation, the follicular phase, ovulation, and the luteal phase, with a normal complete cycle lasting about 28 days from the onset of menstruation. During the follicular phase, the granulosa cells of the follicle produce estradiol, which is essential for the development of the endometrium [1,2]. During the late follicular phase, estrogen levels are high and progesterone levels are low; whereas, during the mid-luteal phase, both estrogen and progesterone levels are high [3].

Typically, the systolic blood pressure (SBP), diastolic BP (DBP), and mean BP (MBP) increase during the luteal phase [4,5]; these changes are related to an increase in the activity of the sympathetic nervous system [6-8]. Increased progesterone levels also affect BP variations during the luteal phase [9,10]. Endometrial and subendometrial vascularization indices change significantly during the menstrual cycle and are characterized by a peak approximately three days before ovulation before decreasing to a nadir five-day post-ovulation [11]. Serial transvaginal ultrasound examinations of normal menstrual cycles revealed that the dominant follicle and ovarian stroma had significantly higher peak systolic blood flow velocity, and the uterine artery's pulsatility index decreased in the mid-luteal phase and was significantly lower than the contralateral side [12]. 

The choroidal blood supply is responsible for 85% of the intraocular blood flow [13], and the sympathetic nervous system primarily regulates the poorly autoregulated choroidal arteries [14-16]. Cold pressor tests can be used to evaluate the sympathetic response in systemic circulatory dynamics [17]. A recent study of choroidal thickness using the cold pressor test with enhanced depth imaging optical coherence tomography (EDI-OCT) in young healthy individuals revealed a significantly diminished subfoveal choroidal thickness (SCT) when sympathetic nerve activity was increased [18]. Additionally, it was noted that mid-luteal phase SCT is dramatically decreased compared with that during the early follicular and ovulatory phases. This decrease was approximately 25 µm less than that in the early follicular phase, suggesting the importance of the menstrual cycle phase in the interpretation of choroidal thickness values in women with a normal menstrual cycle. However, mean arterial pressure did not significantly change during the menstrual cycle [19].

In contrast, the sympathetic nervous system tends to become more hyperactive during the luteal phase than during the follicular phase of the normal menstrual cycle, indicating that changes in systemic hemodynamics cause an increase in choroidal circulation hemodynamics. Furthermore, the rate of change in MBR exhibited a significant positive correlation with changes in DBP and MBP [5]. However, changes in systemic circulatory dynamics and choroidal thickness are poorly understood. This study aimed to investigate the time course of SCT changes during the normal menstrual cycle of healthy Japanese women and its relationship with systemic circulatory dynamics using EDI-OCT.

## Materials and methods

Participants

This prospective study was approved by the Ethics Committee of Fukuoka International University of Health and Welfare (Approval ID: 20-fiuhw-023) and adhered to the tenets of the Declaration of Helsinki. Informed consent was obtained in writing from all participants.

The study included 15 left eyes of 15 healthy Japanese women with normal menstrual cycles who had been examined between May 2022 and January 2023. All the enrolled participants had best corrected visual acuity (BCVA) ≥20/20, no cardiovascular or ophthalmic disease, and were not using any ophthalmic or systemic drugs. We defined normal menstruation as a menstrual cycle of 25-30 days [20]. Each participant underwent assessment once during the late follicular (Days 11 to 15) and mid-luteal phases (Days 20 to 26) [3,5].

All participants were assessed for BCVA, color fundus photography, intraocular pressure (IOP), SBP, DBP, heart rate (HR), and EDI-OCT. The measurement time was set from 12:00 to 15:00 to account for diurnal variance.

EDI-OCT measurements

**Figure 1:**
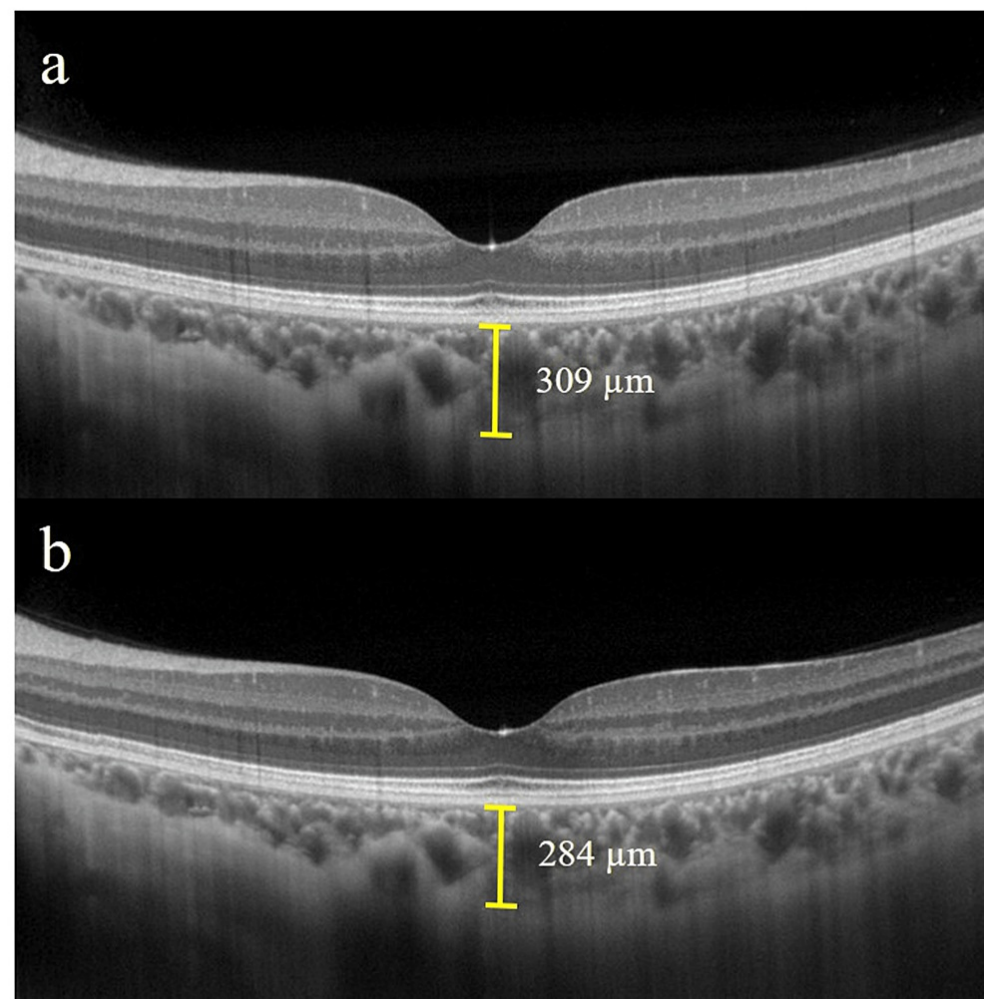
Images of enhanced depth imaging optical coherence tomography (EDI–OCT) in a representative participant (case 4). Horizontal EDI-OCT images of the fovea. The subfoveal choroidal thickness (SCT) was 309.0 µm in the follicular phase (A). In contrast, it decreased to 284.0 µm in the mid-luteal phase (B).

The fovea was horizontally scanned using EDI-OCT (RS-3000 Advance 2; NIDEK, Gakagori, Japan) with a scan length of 12.0 mm. Two examiners (YH and KS) manually measured (using masked participant information) the distance between the outer edge of the high reflectance line corresponding to the retinal pigment epithelium and the outer margin of the choroid to determine the SCT (Figure 1). We assessed the statistical significance of the differences in the average choroidal thickness values between stages.

IOP, BP, and HR measurements

IOP, SBP, DBP, and HR were assessed at every evaluation point for each participant. We calculated MBP from SBP and DBP according to the formula given below [16]:

 MBP = DBP + 1/3(SBP-DBP) (1)

Statistics

All data were presented as mean ± SD (standard deviation). For determining the changes in IOP, SBP, DBP, MBP, HR, and SCT, the Wilcoxon signed rank test was performed. For all tests, p-values < 0.05 for all tests were considered statistically significant.

## Results

The clinical characteristics of all individuals are summarized in Table 1. In total, 15 healthy women (mean age: 20.2 ± 0.8 years) were included.

**Table 1:** Characteristics and changes in intraocular pressure and systemic factors at late follicular phase and mid-luteal phase. SD, standard deviation; SE, spherical equivalent; D, diopter; AL, axial length; IOP, intraocular pressure; FP, follicular phase; LP, luteal phase; SBP, systolic blood pressure; DBP, diastolic blood pressure; MBP, mean blood pressure; HR, heart rate; bpm, beat per minutes; SCT, subfoveal choroidal thickness

Case	Age	SE (D)	AL (mm)	IOP (mmHg)		SBP (mmHg)		DBP (mmHg)		MBP (mmHg)		HR (bpm)		SCT (µm)	
				Late FP	Mid-LP	Late FP	Mid-LP	Late FP	Mid-LP	Late FP	Mid-LP	Late FP	Mid-LP	Late FP	Mid-LP
1	20	-0.5	23.2	18.0	17.3	115.0	117.0	76.5	75.0	89.4	89.0	85.5	89.5	301.0	282.0
2	20	-5.1	26.4	14.3	14.3	94.5	94.5	54.0	68.0	67.5	76.9	76.5	73.5	153.0	149.0
3	20	-9.8	27.0	12.7	15.0	112.0	125.0	76.5	80.5	88.4	95.3	75.5	77.0	161.0	155.5
4	19	-0.8	24.0	14.3	14.0	90.5	105.0	55.0	72.0	66.9	83.0	65.0	65.0	309.0	284.0
5	19	-1.5	23.2	19.7	17.3	101.5	111.0	64.5	62.5	76.8	78.7	72.0	76.5	352.0	339.0
6	19	-1.6	23.4	8.7	10.7	94.5	97.0	56.5	60.0	69.2	72.4	75.5	70.0	247.0	260.0
7	20	-6.2	26.0	17.0	17.7	93.0	99.5	58.0	65.5	69.7	76.8	58.0	58.0	180.0	167.0
8	20	-7.5	24.5	12.7	14.0	101.5	111.0	61.5	70.0	74.9	93.0	77.5	93.0	382.5	370.0
9	20	-5.2	26.9	12.7	14.7	98.5	115.0	55.5	63.0	69.8	78.3	70.5	78.0	235.0	223.0
10	22	+1.1	23.8	12.0	10.0	102.0	100.0	74.0	63.5	83.3	75.6	68.5	68.0	256.0	268.0
11	21	-5.8	26.7	9.3	10.0	107.5	100.0	69.0	67.5	81.8	78.4	70.0	80.0	215.0	213.0
12	20	-4.5	25.6	12.7	14.3	92.5	116.0	60.0	75.5	70.9	89.0	87.0	82.0	210.5	216.5
13	21	-5.7	25.3	14.0	15.3	118.0	114.5	73.5	73.5	88.3	87.2	67.0	69.0	255.5	241.0
14	21	-1.7	23.3	19.7	18.3	86.5	114.5	58.5	71.0	67.7	85.5	84.0	103.5	204.0	194.0
15	21	-4.7	25.7	13.7	12.3	101.0	114.0	69.0	79.5	79.7	91.0	72.0	81.5	278.0	268.0
Mean ± SD	20.2 ± 0.8	-4.0 ± 3.0	25.0 ± 1.4	14.1 ± 3.2	14.3 ± 2.6	100.6 ± 9.2	108.9 ± 8.9	64.1 ± 8.2	69.8 ± 6.2	76.3 ± 8.2	83.3 ± 7.1	73.6 ± 7.9	77.6 ± 11.5	249.3 ± 66.6	242.0 ± 63.6

**Table 2:** Comparison of intraocular pressure and systemic factors at late follicular phase and mid-luteal phase. IOP, intraocular pressure; SBP, systolic blood pressure; DBP, diastolic blood pressure; MBP, mean blood pressure; HR, heart rate; bpm, beat per minutes; SCT, subfoveal choroidal thickness Wilcoxon signed-rank test

	Late follicular phase	Mid-luteal phase	P-value
IOP (mmHg)	14.1 ± 3.2	14.3 ± 2.6	0.593
SBP (mmHg)	100.6 ± 9.2	108.9 ± 8.9	0.010
DBP (mmHg)	64.1 ± 8.2	69.8 ± 6.2	0.020
MBP (mmHg)	76.3 ± 8.2	83.3 ± 7.1	0.012
HR (bpm)	73.6 ± 7.9	77.6 ± 11.5	0.074
SCT (µm)	249.3 ± 66.6	242.0 ± 63.6	0.033

IOP, BP, and HR outcomes

The IOP, BP, and HR changes are summarized in Tables 1 and 2, respectively. SBP, DBP, and MBP were significantly elevated in the late mid-luteal phase compared to the follicular phase (p = 0.010, p = 0.020, and p = 0.012, respectively) (Table 2). Conversely, there were no significant changes in IOP or HR (p = 0.593 and p = 0.074 respectively) (Table 2).

EDI-OCT data

SCT changes are summarized in Tables 1 and 2. The mean value of SCT in the late follicular was 249.3 ± 66.6 μm and that in mid-luteal phases was 242.0 ± 63.6 μm. SCT was significantly lower in the mid-luteal phase compared to the late follicular phase (p = 0.033) (Table 2).

## Discussion

In the present study, significant decreases in choroidal thickness at the fovea in the mid-luteal phase were found to be associated with increases in systemic circulatory factors. Sympathetic activity was observed to increase during the luteal phase of the normal menstrual cycle, indicating that choroidal thickness decreases with increased systemic hemodynamics.

In a previous study of macular choroidal circulation hemodynamic in young healthy adult women with normal menstrual cycles, choroidal blood flow velocity was found to increase in the luteal phase, when sympathetic activity is predominant, with an increase in systemic circulatory activity. In addition, choroidal blood flow velocity showed a statistically significantly positive correlation with DBP and MBP [5]. There are suggestions about the relationship between choroidal circulatory dynamics and changes in the systemic circulatory system during the menstrual cycle, but little is known about the connection between choroidal morphology and systemic circulatory dynamics. The SCTs during the early follicular, ovulatory, and mid-luteal phases were 383.8, 373.7, and 359.0 μm, respectively, with thinnest in the mid-luteal phase. Specifically, in young healthy women, choroidal thickness decreased significantly in the mid-luteal phase of the menstrual cycle, as observed in our study. However, the mean arterial pressure did not differ significantly during the menstrual cycle [19]. Besides, the activity of the autonomic nervous system varies during menstruation. It has been reported that in young women, sympathetic activity increases and parasympathetic activity decreases during the luteal phase [6-8]. It is known that estrogen levels are high and progesterone levels are low in the late follicular phase, and both levels are high in the mid-luteal phase [4]. Although fluctuations in ovarian hormones, such as estrogen and progesterone, cause these changes, each of them affects the autonomic nervous system. Additionally, it has been demonstrated that an elevation in progesterone increases BP [9,10]. These mechanisms suggest that systemic circulation is increased in the luteal phase [3-10]. 

According to a report investigating vascular reactivity in the retinal choroid with functional OCT, vascular perfusion density decreased in the choroid but did not change in the retina during a cold pressor test performed in healthy subjects [15]. In addition, after the cold pressor test, SCT was reported to decrease with an increase in SBP, DBP, MBP, and ocular perfusion pressure owing to increased sympathetic nerve activity [18]. Also, the diurnal variation in SCT is significant, decreasing most at 6:00 PM, when the sympathetic nervous system is active [21].

Therefore, in the mid-luteal phase, choroidal thickness may decrease due to a reduction in choroidal volume resulting from vasoconstriction caused by increased sympathetic activity, which is associated with an increase in systemic circulatory activity.

This study has several limitations. First, SCT was measured manually using a B-scan of EDI-OCT, which may be influenced by measurement bias. More investigations are required for automatic measurement of the volume and thickness of the retina and choroid utilizing C-scans with swept-source OCT in order to overcome this bias. Second, to evaluate the relationship between choroidal function and morphology in more detail, OCT angiography should also be used. Furthermore, the sample size was small with only two phases investigated (follicular and luteal phases); future research needs to increase the sample size and investigate changes in choroidal morphology in other phases of the menstrual cycle.

## Conclusions

The results of the present study suggest that choroidal thickness decreases during the mid-luteal phase of normal menstrual cycles in young, healthy Japanese women, depending on the systemic circulation dynamics. EDI-OCT is a potentially useful noninvasive quantitative method of assessing choroidal and systemic changes during the normal menstrual cycle. As the change in SCT was only approximately 7 µm, the menstrual cycle of Japanese women may have little influence on the interpretation of choroidal thickness data in clinical practice. In the future, the relationship between choroidal thickness and systemic circulatory dynamics in participants with irregular menstrual cycles or premenstrual syndrome should be analyzed in detail, and the results of this study may provide baseline data for comparisons with the results of such analyses.
